# Supplementary material for: SARS-CoV-2 Infection in Pregnancy: Placental Histomorphological Patterns, Disease Severity and Perinatal Outcomes
Source: Int J Environ Res Public Health. 2022 Aug 3;19(15):9517. doi: 10.3390/ijerph19159517 (PMC9368100; doi:10.3390/ijerph19159517)
Supplement: Supplementary file 1 [file ijerph-19-09517-s001.zip › ijerph-1824956-supplementary.pdf]

Supplementary Table S1. Clinical spectrum of SARS-CoV-2 infection.

**Clinical Spectrum of COVID-19**	**Diagnostic Criteria**
Asymptomatic	Individuals who are tested positive for SARS-CoV-2 using a virologic test (including nuclei acid amplification test or an antigen test) but lacking symptoms that of COVID-19
Mild illness	Individuals who have some symptoms and signs of COVID-19 (such as fever, cough, sore throat, malaise, nausea, vomiting, headache, muscle pain, diarrhoea, loss of taste and smell) but without clinical or radiological evidence of viral pneumonia.
Moderate illness	Individuals who have clinical or radiological signs of lower respiratory disease (such as fever, cough, shortness of breath, and dyspnoea) and with oxygen saturation (SpO_2) ≥ 94% on room air at sea level.
Severe illness	Individuals who have clinical or radiological signs of lower respiratory disease (such as fever, cough, shortness of breath, and dyspnoea) plus one of the following: a respiratory rate >30 breaths/min, severe respiratory distress or SpO_2 < 94% on room air at sea level or lung infiltrates >50%.
Critical illness	Individuals who have respiratory failure, septic shock and/or multiple organ dysfunction.

Supplementary Table S2. Histological features of placentas delivered from symptomatic vs asymptomatic COVID-19-infected cases.

**Histological features**		**Cases**		***p* value**
		**Asymptomatic n = 19 (%)**	**Symptomatic n = 28 (%)**	
Maternal vascular malperfusion	Accelerated villous maturation/ distal villous hypoplasia	1	6	0.215
	Syncytial knots (mean ± SD)	33.56 ± 13.81	34.56 ± 11.87	0.479
	Villous infarction	3	3	0.674
	Decidual vasculopathy	8	14	0.767
	Decidual arterial thrombosis	6	3	0.129
Fetal vascular malperfusion	Avascular villi	0	3	0.262
	Fetal vascular thrombosis	0	3	0.262
	Chorangiosis	5	2	0.102
Chronic inflammation	Chronic deciduitis	1	4	0.396
	Chronic villitis, low grade	1	4	0.264
	Chronic villitis, high grade	1	5	
	Chronic histiocytic intervillositis	2	8	0.168
Maternal inflammatory response	Acute subchorionitis	8	13	1.000
	Acute chorioamnionitis	1	1	
Fetal inflammatory response	Chorionic vasculitis &/ umbilical phlebitis	1	2	1.000
Others	Oedematous villi	1	4	0.635

Supplementary Table S3. Logistic regression analysis of risk factors for adverse maternal and neonatal outcomes in COVID-19-infected pregnancies.

**Factors**		**Adverse Maternal and Neonatal Outcomes**										
			**Preterm delivery**		**Neonatal death**		**Poor Apgar scores**		**SGA**		**Maternal death**	
		**Total cases**	**Crude OR (95% CI)**	***p* value**	**Crude OR (95% CI)**	***p* value**	**Crude OR (95% CI)**	***p* value**	**Crude OR (95% CI)**	***p* value**	**Crude OR (95% CI)**	***p* value**
**Clinical Characteristics**												
Mother's age		47	1.020 (0.895, 1.162)	0.768	1.151 (0.896, 1.477)	0.271	1.124 (0.943, 1.340)	0.191	0.937 (0.821, 1.070)	0.335	1.069 (0.895, 1.329)	0.551
COVID-19 status	Symptomatic	28	3.250 (0.860, 12.284)	0.082	193856978.1 (0.000)	0.998	538491582.8 (0.000)	0.998	2.000 (0.610, 6.553)	0.252	269245805.6 (0.000)	0.998
	Asymptomatic	19	Reference									
COVID-19 status	Active	33	5.000 (0.964, 25.939)	0.055	161547490.5 (0.000)	0.999	43935496.7 (0.000)	0.999	3.600 (0.970, 13.357)	0.056	222824124.6 (0.000)	0.999
	Recover	14	Reference									
COVID-19 status	Severe-critical	11	15.750 (2.815, 88.123)	**0.002***	605803083.4 (0.000)	0.998	2827080938 (0.000)	0.997	2.386 (0.543, 10.476)	0.249	13.125 (1.203, 143.233)	**0.035***
	Asymptomatic, mild, moderate	36	Reference									
Maternal comorbid	1 comorbid	14	1.389 (0.327, 5.898)	0.656	1.538 (0.088, 26.821)	0.768	0.462 (0.043, 4.952)	0.523	0.800 (0.173, 3.690)	0.775	0.000 (0.000)	0.999
	More than 1 comorbid	12	2.500 (0.572, 10.932)	0.224	1.818 (0.103, 31.996)	0.683	2.000 (0.334, 11.969)	0.448	5.000 (0.942, 26.530)	0.059	1.900 (0.232, 15.582)	0.550
	No comorbid	21	Reference	0.475		0.915				0.044		0.836
Caesarean section	Yes	37	6.857 (0.786, 59.814)	0.081	142541891.5 (0.000)	0.999	376944125.6 (0.000)	0.999	N/A	N/A	195815142.2 (0.000)	0.999
	No	10	Reference									
**Placental Histomorphological Features**												
MIR	Yes	24	6.500 (1.679, 25.162)	0.007*	242321235.8 (0.000)	0.998	8.118 (0.892, 73.841)	0.063	1.077 (0.339, 3.424)	0.900	1.048 (0.135, 8.131)	0.965
	No	23	Reference									
FIR	Yes	3	1017150600 (0.000)	0.999	118205475.4 (0.000)	0.999	305630381.0 (0.000)	0.999	2565754162 (0.000)	0.999	161547505.6 (0.000)	0.999
	No	44	Reference									

Villitis and Intervillositis	Yes	12	1.369 (0.357, 5.245)	0.647	1.500 (0.124, 18.193)	0.750	1.200 (0.201, 7.182)	0.842	1.500 (0.401, 5.605)	0.547	208448366.7 (0.000)	0.999
	No	35	Reference									
Decidual vasculopathy	Acute atherosis	5	1.185 (0.166, 8.471)	0.866	0.000 (0.000)	0.999	0.000 (0.000)	0.999	6.000 (0.582, 61.842)	0.132	0.000 (0.000)	0.999
	Retention of SM	17	0.970 (0.268, 3.512)	0.963	0.000 (0.000)	0.998	0.198 (0.022, 1.820)	0.152	0.818 (0.228, 2.933)	0.758	0.000 (0.000)	0.998
	No	25	Reference									
MVT	Yes	9	1.538 (0.352, 6.730)	0.567	138469271.6 (0.000)	0.999	1.886 (0.302, 11.772)	0.497	3.150 (0.578, 17.167)	0.185	1.458 (0.134, 15.915)	0.757
	No	38	Reference									
Accelerated villous maturation	Yes	21	2.468 (0.730, 8.344)	0.146	269245816.3 (0.000)	0.998	10.000 (1.095, 91.309)	**0.041***	1.393 (0.432, 4.490)	0.579	1.263 (0.163, 9.815)	0.823
	No	26	Reference									
Avascular villi/FVT	Yes	3	1.143 (0.096, 13.617)	0.916	118205478.0 (0.000)	0.999	3.167 (0.247, 40.564)	0.376	1.520 (0.128, 18.032)	0.740	6.833 (0.473, 98.811)	0.159
	No	44	Reference									
Chorangiosis	Yes	7	4.000 (0.439, 36.444)	0.219	0.000 (0.000)	0.999	0.000 (0.000)	0.999	4.167 (0.717, 24.227)	0.112	0.000 (0.000)	0.999
	No	40	Reference									

Abbreviations: CI – confidence interval, FIR – fetal inflammatory response, FVT – fetal vascular thrombosis, OR – odds ratio, MIR – maternal inflammatory response, MVT – maternal vascular thrombosis, N/A – not analysed, SGA – small for gestational age, SM – smooth muscle, *statistically significant
